# Editorial: AI and data science in pulmonary and critical care physiology and medicine

**DOI:** 10.3389/fphys.2024.1375414

**Published:** 2024-03-05

**Authors:** Yuh-Chin Huang, Paresh Giri, Octavian Ioachimescu, An-Kwok Ian Wong

**Affiliations:** ^1^ Department of Medicine, Duke University Medical Center, Duke University, Durham, NC, United States; ^2^ Department of Medicine, Loma Linda University, Loma Linda, CA, United States; ^3^ Department of Medicine, Emory University, Atlanta, GA, United States

**Keywords:** machine learning, sepsis, ARDS, lung volumes, ventilator-associated complication, DVT

Recent advances in computational power provide us new opportunities to refine clinical and physiological assessment in pulmonary and critical care medicine. This Research Topic looks for studies that leverage AI and data science in the identification, interpretation, prediction, and management of physiologic and/or pathologic processes. In total, five articles were selected.

The article by Qin et al. used data from 502 patients to develop a nomogram model in combination with thromboelastography (TEG) to predict the development of venous thromboembolism (VTE) after lung cancer surgery. The prediction model was constructed using univariate and multivariate logistic regression analyses using variables of FEV1, age, operation duration, and postoperative TEG parameters k value(K) and reaction time(R) and had the following equation: Logit(P) = 5.278 + 0.088 × Age-0.928 × FEV1 + 0.017 × Operation duration-3.05 × Post-K-1.345 × Post-R. The nomogram model demonstrated better predictive power (AUC 0.913) than the modified Caprini model (AUC 0.681), with the consistency index (C-index) being greater. If validated in other data sets, the model could provide individual risk prediction for patients after lung cancer surgery and has the potential to help clinicians develop individualized preventive anticoagulation strategies to reduce the incidence of DVT.

The article by Rui et al. aimed to explore the clinical application of an AI-3D reconstruction system in measuring lung volume and analyze its practical value in donor-recipient size matching in lung transplantation. The study compared lung volume calculated from an AI-3D reconstruction system (AI-3DCTVol) with the predicted TLC (pTLC) and actual TLC (aTLC) measured by PFT in 75 Chinese subjects. Overall, they found a good correlation between AI-3DCTVol and aTLC (the intraclass correlation [ICC] 0.79 [95% CI: 0.68–0.87]). The AI-based 3D reconstruction of lung parenchyma showed good performance, with potential future application in lung volume assessments before lung transplantation. The sample size, however, was small. The results will need to be validated in a larger cohort and in different racial and ethnic groups.

The article by Tan et al. described a machine learning model that can predict respiratory decompensation based on the CDC definition of ventilator-associated complication (VAC) using the MIMIC-III database. A VAC is defined as sustained hypoxemia (an increase in daily minimum PEEP ≥3 cm H_2_O or FiO_2_ ≥ 0.20 sustained for at least 2 calendar days following a baseline period (2 calendar days) of stability or improvement) plus altered leukocyte count (≥12,000 cells/mm^3^ or ≤4,000 cells/mm^3^) and/or temperature (>38°C or <36°C), a new antimicrobial prescription has been started and sustained for at least 4 calendar days by the attending physician. The authors constructed random forest models to predict occurrence of VAEs, then trained machine learning models on two separate populations: Cohort One −1921 patients with sufficient data for statistical feature generation and Cohort Two-464 patients with sufficient physiologic time series data. Discrimination of occurrence of VAEs was possible with AUROC as high as 0.83 suggesting that such prediction models could, with further methodological finesse, complement clinical workflow in future. Strengths of the study include rigorous methodology including use of low and high frequency features in two patient populations. Testing the model in low and high frequency sampling datasets help select an optimal time step, in this study, the authors found the best results using the data 36 h before VAC onset. Limitations of the study included data preprocessing, such as replacement of missing data and down sampling and the need for validation in additional dataset. Importantly, the CDC/NHSN working group VAC definition that was used in the study is not a clinical definition and is not intended for use in the clinical management of patients. Also, MIMIC-III contains data from 2001 to 2012. Since then, the ventilator management has continued to be optimized which would affect the incidence of VAC. The results will need to be validated using more recent data.

The article by Wang et al. investigated whether or not early albumin supplementation could reduce 28-day mortality of ARDS associated with septic shock. They retrospectively analyzed the MIMIC-III database and compared the group that received human albumin with the non-albumin group using propensity score matching to minimize the bias. They found the 28-day mortality in the albumin group was lower (34.8%, vs. 48.1%, *p* = 0.031). The benefit of human albumin treatment appeared to be more pronounced in patients with a SOFA score of ≤10. The study did not use any AI technique but was included because it used the large MIMIC-III database and thus was considered data science research. The mechanism for the better outcome could be related to higher fluid intake and thus better hemodynamic support in the albumin group. Whether the results can be extended to ARDS associated with other causes is unclear. As mentioned above, the MIMIC-III database contained data from 2001 to 2012. The management of septic shock and ARDS has continued to evolve since then. So, results derived from MIMIC-III database need to be interpreted with caution.

The article by Bai et al. sought to build a machine learning diagnostic model for patients with sepsis-associated ARDS to identify clinical phenotypes in this population, and explore the differences among these phenotypes. The data were extracted from MIMIC-IV with models built based on 19,249 sepsis patients and 5,947 sepsis-associated ARDS patients. They found the AdaBoost (Decision Tree) model achieved the best performance with an area under the receiver operating characteristic curve (AUC) of 0.895 with an accuracy of 70.06%, a sensitivity of 78.11% and a specificity of 78.74%. They also identified three clinical clusters. Cluster 0 had lower mortality and milder laboratory abnormalities while cluster 1 had higher mortality and significant laboratory abnormalities. Cluster 2 was more complex with intermediate mortality and longer ICU stay. The results were validated in the test set. In the editorial team’s opinion, the identification of cluster 2 that had long ICU stay and moderate mortality was important. This was the group that presented the most uncertainly to the intensivist clinically and deserved more granular analysis in the future to further define the survivability after a long ICU stay.

As AI will continue to evolve rapidly, more AI models for different diseases and conditions will be published. The readers should be aware of the limitations of each study and make proper judgements.

